# Tailoring morphology of cobalt–nickel layered double hydroxide via different surfactants for high-performance supercapacitor

**DOI:** 10.1098/rsos.180867

**Published:** 2018-09-12

**Authors:** Bangqing Xiao, Wenliang Zhu, Zhong Li, Jiliang Zhu, Xiaohong Zhu, Giuseppe Pezzotti

**Affiliations:** 1 College of Materials Science and Engineering, Sichuan University, Chengdu 610064, People's Republic of China; 2 Ceramic Physics Laboratory, Kyoto Institute of Technology, Sakyo-ku, Matsugasaki, 606-8585 Kyoto, Japan

**Keywords:** cobalt–nickel layered double hydroxide, surfactant, supercapacitor

## Abstract

Tailoring the morphology of cobalt–nickel layered double hydroxide (LDH) electrode material was successfully achieved via the process of cathodic electrodeposition by adding different surfactants (hexamethylenetetramine, dodecyltrimethylammonium bromide (DTAB) or cetyltrimethylammonium bromide). The as-prepared Co_0.75_Ni_0.25_(OH)_2_ samples with surfactants exhibited wrinkle-like, cauliflower-like or net-like structures that corresponded to better electrochemical performances than the untreated one. In particular, a specific capacitance of 1209.1 F g^−1^ was found for the cauliflower-like Co_0.75_Ni_0.25_(OH)_2_ electrode material using DTAB as the surfactant at a current density of 1 A g^−1^, whose structure boosted ion diffusion to present a good rate ability of 64% with a 50-fold increase in current density from 1 A g^−1^ to 50 A g^−1^. Accordingly, the asymmetric supercapacitor assembled by current LDH electrode and activated carbon electrode showed an energy density as high as 21.3 Wh kg^−1^ at a power density of 3625 W kg^−1^. The relationship between surfactant and electrochemical performance of the LDH electrode materials has been discussed.

## 1. Introduction

Climate change and fossil fuel depletion have triggered intense scientific research to explore renewable and sustainable energy sources; demands from hybrid electric vehicles and pulsed power systems also promote the development of alternative energy [1–4]. The supercapacitor is one of the promising devices to meet the ever-growing need, with its higher energy density than that of common capacitors and better power density than that of batteries [2].

Exploring electrode materials with high specific capacitance (Sc) is necessary to improve the energy density of supercapacitors. Carbon materials, conducting polymers and transition metal oxides/hydroxides with high surface area are commonly used as supercapacitor electrode materials [3]. Carbon-based materials (carbon aerogels, carbon nanotubes and graphene) [4–6] as electrode materials for electrical double-layer capacitors have been extensively studied to increase their low Sc which directly limited their practical application [7]. Conducting polymers (polyaniline, polypyrrole, poly(3,4-ethylenedioxythiophene), etc.) have significant drawbacks of low rate of charge–discharge due to slow ion diffusion within the bulk of the electrode [8]. Transition metal oxides [9]/hydroxides [10] /sulfides [11] are promising candidates owing to their low environmental toxicity and high Sc, which have been widely investigated in an attempt to attain high Sc and long cycle life. Among these materials, layered double hydroxide (LDH) with the chemical formula ${\left[{\text{M}}_{1-x}^{2+}{\text{M}}_{x}^{3+}{\left(\text{OH}\right)}_{2}]{\;}^{x+}[A_{x/n}\right]}^{n-}∙\text{m}{\text{H}}_{2}\text{O}$ (where M^2+^ is a divalent ion including Ni^2+^, Zn^2+^, Mn^2+^; M^3+^ is a trivalent ion, i.e. Co^3+^, Al^3+^, Cr^3+^; ${\text{A}}_{x/n}^{n-}$ represents anion $\text{C}{\text{O}}_{3}^{2-}$, OH^−^, $\text{N}{\text{O}}_{3}^{-}$, $\text{S}{\text{O}}_{4}^{2-}$ etc.) [12] has never ceased to attract research interest arising from a lamellar structure and large interlayer spacing with abundant active area [13]. Nickel- and cobalt-based LDHs are significant branches among these LDH materials because of their superior theoretical Sc [14].

There are various techniques used to fabricate LDH electrode materials such as co-deposition [15], hydrothermal [16], microwave method [17], vacuum freeze-drying technique [18] and electrochemical deposition [19]. Among these methods, the electrodeposition method possesses several advantages: (i) electrodeposition route offers flexibility to control weight and thickness of the film; (ii) the active materials are deposited on the substrate directly, avoiding decrease in conductivity caused by the resistance of the binder; and (iii) electrodeposition offers a facile and economical approach to large area deposition, which is one of the crucial factors for commercial application. It has been well demonstrated that nanostructure shows admirable rate ability and capability compared with common materials [20], and adding a surfactant to modify the morphology of the LDH material benefits the control of nanostructure during the process of electrodeposition [21]. The effects of surfactant depend on the interactions with precursor; the surfactant head/hydrocarbon tail could lead to new orders at the interface and/or inside the system to some extent [22]. Meanwhile, the surfactant can lower the surface tension of the solution [23].

In this paper, we proposed a facile route to synthesize cobalt–nickel LDH films of different morphologies by electrodepositing from aqueous solution in the presence of different surfactants (hexamethylenetetramine (HMT), dodecyltrimethylammonium bromide (DTAB) or cetyltrimethylammonium bromide (CTAB)). Inductively coupled plasma–optical emission spectrometry (ICP), X-ray diffraction (XRD), Fourier transform infrared (FTIR) spectroscopy, scanning electron microscopy (SEM) and transmission electron microscopy (TEM) were applied to investigate the structures and morphologies of the as-prepared samples. Asymmetric supercapacitors (ASCs) based on current cobalt nickel LDHs as positive electrodes and activated carbon (AC) as negative electrodes were fabricated. The electrochemical performances of the ASC devices were also investigated.

## 2. Material and methods

### 2.1. Materials

Nickel nitrate hexahydrate (Ni(NO_3_)_2_·6H_2_O), cobalt nitrate hexahydrate (Co(NO_3_)_2_·6H_2_O), thiourea (CH_4_N_2_S) and HMT were acquired from China National Medicine Corporation Ltd. DTAB and CTAB were purchased from Chengdu Kelong Chemical Regent Co. Ltd (China). All chemicals were of analytical grade and used without further purification. The nickel foam was washed in 6M HCl and acetone with ultrasonication for 10 min, then washed with de-ionized water and alcohol several times, and dried in an oven at 60°C. The nickel foam substrate was rolled to a thickness of 0.2 mm before use.

### 2.2. Synthesis of electrodes

Electrochemical cathodic deposition was carried out for the synthesis of the LDH samples in an aqueous solution; the solution was prepared by dissolving 2.5 mM Ni(NO)_2_·6H_2_O, 7.5 mM Co(NO)_2_·6H_2_O, 2 mM CH_4_N_2_S with vigorous stirring, without or with 2 mM HMT, DTAB or CTAB. The samples were prepared firstly by using a multi-potential-step technique in a three-electrode configuration with Ag/AgCl as the reference electrode, platinum as the counter electrode and nickel foam as the working electrode at −2 V for 50 s under constant magnetic stirring. The obtained samples were rinsed with de-ionized water and dried at 60°C for several minutes. Then the above procedure was repeated five times. The precursors were annealed at 200°C for 2 h. The mass of active material was determined by the weight difference of the nickel foam substrate before and after deposition, and the mass loading of active material was about 0.6–1 mg per square centimetre.

### 2.3. Characterization and measurements

The composition and structure of the samples were characterized by ICP (ARCOS SOP), FTIR spectroscopy (Nicolet 6700), XRD (DX-2700X, Cu K*α* radiation *λ* = 0.15406 nm), SEM (JSM-7500F), TEM (JEM-2010) and X-ray photoelectron spectroscopy (XPS) (XSAM 800). The electrochemical performance was measured by electrochemical workstation (CHI660E) and LAND test system (CT2001A).

## 3. Results and discussion

### 3.1. Compositions and growth mechanism

Table 1 shows the content of the metal ions measured by ICP in the synthesized LDH samples. The mole ratio of cobalt/nickel in the composites is very close to 3 : 1, indicating that the relative ratio is in good agreement with the initial ratio in the aqueous solution. As the mole ratio is 3 : 1, the obtained electrode materials can be denoted as Co_0.75_Ni_0.25_(OH)_2_ (no surfactant), Co_0.75_Ni_0.25_(OH)_2_: H (HMT as surfactant), Co_0.75_Ni_0.25_(OH)_2_: D (DTAB as surfactant) and Co_0.75_Ni_0.25_(OH)_2_: C (CTAB as surfactant).

**Table 1.** Concentration of metal elements in the samples.

samples	Co (µg ml^−1^)	Ni (µg ml^−1^)	Co (mM l^−1^)	Ni (mM l^−1^)	Co/Ni
Co_0.75_Ni_0.25_(OH)_2_	51.0	17.3	0.86	0.29	2.96
Co_0.75_Ni_0.25_(OH)_2_: H	125	40.8	2.11	0.69	3.06
Co_0.75_Ni_0.25_(OH)_2_: D	67.8	23.5	1.15	0.40	2.88
Co_0.75_Ni_0.25_(OH)_2_: C	73.6	24.0	1.24	0.41	3.02

The cathodic electrodeposition of the Co_0.75_Ni_0.25_(OH)_2_ LDHs includes an electrochemical reaction and subsequent precipitation of mixed hydroxide, as described by the following two equations:

3.1 $$\text{N}{\text{O}}_{3}^{-}+7{\text{H}}_{2}\text{O}+8e^{-}\to \;\text{N}{\text{H}}_{4}^{+}+10\text{O}{\text{H}}^{-}$$

and

3.2 $$x\text{C}{\text{o}}^{2+}+(1-x)\text{N}{\text{i}}^{2+}+2\text{O}{\text{H}}^{-}\to \text{C}{\text{o}}_{x}\text{N}{\text{i}}_{1-x}{\left(\text{OH}\right)}_{2}.$$

$\text{N}{\text{O}}_{3}^{-}$ is reduced on the cathode to produce hydroxide ions, and the increment of pH value promotes uniform precipitation of mixed (Ni, Co) hydroxide on the surface, considering that the solubility product constant (*K*_sp_) at room temperature of Co(OH)_2_ (2.5 × 10^−16^) is close to that of Ni(OH)_2_ (2.8 × 10^−16^) [24].

**Figure 1.**
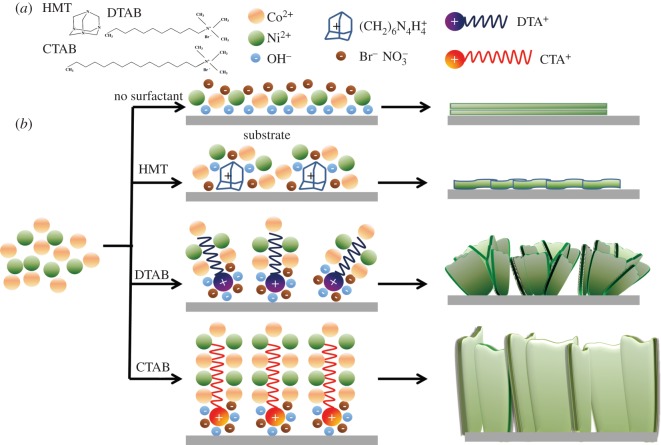
(*a*) Molecular structures of selected surfactants; (*b*) the possible formation mechanism of Co_0.75_Ni_0.25_(OH)_2_ LDHs supported on the nickel foam substrate.

The possible formation mechanism of Co_0.75_Ni_0.25_(OH)_2_ LDHs supported on Ni foams is shown in figure 1. Under the electrodeposition condition, HMT probably hydrolyses in the presence of distilled water to form the complex ${\left(\text{C}{\text{H}}_{2}\right)}_{6}{\text{N}}_{4}{\text{H}}_{4}^{+}$ and generates OH^−^ ions [25,26]. Meanwhile, DTAB and CTAB ionize and provide extra ions, thus enhancing the conductivity of the aqueous solution. The surfactant cations (${\left(\text{C}{\text{H}}_{2}\right)}_{6}{\text{N}}_{4}{\text{H}}_{4}^{+}$, DTA^+^, CTA^+^) can be electrostatically adsorbed on the negatively charged surface of working electrode and expected to serve as templates [27]. The different structures of three surfactants play key roles in forming the morphology of electrode materials. As shown in figure 1*a*, ${\left(\text{C}{\text{H}}_{2}\right)}_{6}{\text{N}}_{4}{\text{H}}_{4}^{+}$ is a compound with a cage-like structure [28], and relatively uniform winkles are formed. Compared with CTA^+^, DTA^+^ has a shorter carbon chain length [29], so the single nano-sheet length (100–200 nm) of Co_0.75_Ni_0.25_(OH)_2_: D is shorter than that (500–600 nm) of Co_0.75_Ni_0.25_(OH)_2_: C (figure 5*c*,*d*), which tends to aggregate to reduce the surface energy from a thermodynamic perspective [30]. Thus, the smaller nano-sheets are relatively inclined to form a three-dimensional (3D) flower-like structure by self-assembling.

### 3.2. Structure measurements and morphology analysis

**Figure 2.**
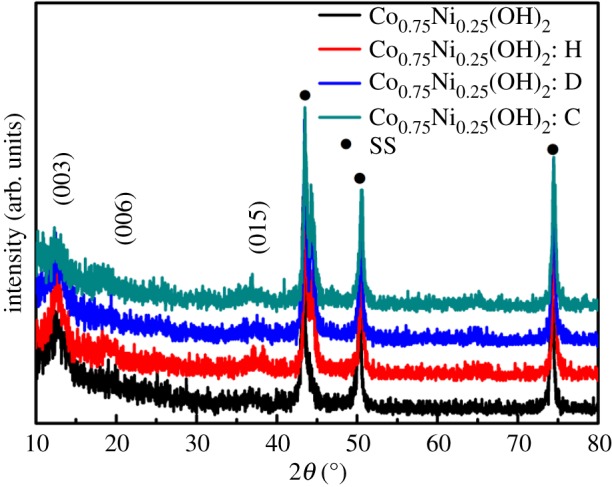
XRD patterns of as-prepared pristine Co_0.75_Ni_0.25_(OH)_2_ and Co_0.75_Ni_0.25_(OH)_2_ electrodes synthesized by using different surfactants on stainless steel substrates.

XRD analysis is a powerful tool to clarify the structure of the as-electrodeposited sample in the 2*θ* range of 10°–80°. The background peaks of nickel foam are very strong, which would disturb the diffraction peaks of electrode materials; so, cleaned stainless steel was employed to act as a substrate. As shown in figure 2, there are three strong diffraction peaks of stainless steel substrate (SS). The diffraction peaks at 2*θ* = 11.1°, 22.2°, 38.2° are indexed as (003), (006) and (015), which originate from the characteristic lattice planes of hydrotalcite-like LDH phase [14,31]. The small thickness and polycrystalline nature bring about the weak diffraction intensities of the cobalt–nickel LDH films [32,33].

**Figure 3.**
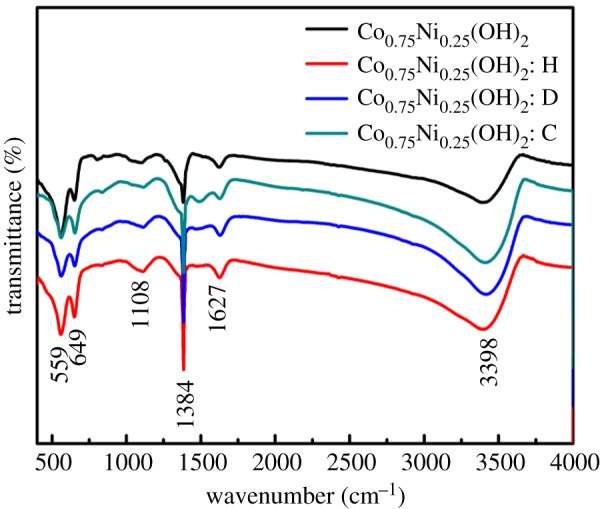
FTIR spectra of as-prepared pristine Co_0.75_Ni_0.25_(OH)_2_ and Co_0.75_Ni_0.25_(OH)_2_ electrodes synthesized by using different surfactants.

Figure 3 presents the FTIR spectra of the as-prepared samples. The stretching vibrations of Co–O and Ni–O result in the absorption bands at 559 cm^−1^ and 649 cm^−1^, respectively [34]. The weak band at 1108 cm^−1^ is due to a small amount of intercalated sulfate [31]. It is convinced that sulfate exhibits better conductivity than carbonate, indicating superior electrochemical performance [35]. The band at 1483 cm^−1^ is contributed by carbonate resulting from the adsorption of atmospheric CO_2_ and the intercalation of carbonate caused by electrodeposition process from the electrolyte dissolving CO_2_ in air. There is a sharp and intense band at 1384 cm^−1^ corresponding to the $\text{N}{\text{O}}_{3}^{-}$ derived from the intercalation of nitrate ions in the layered structure [19]. The bands at 3398 cm^−1^ and 1627 cm^−1^ can be assigned to stretching vibration and deformation vibration of the adsorbed water molecules as well as the interlayer water molecules [36]. These data shed light on the formation of a layered structure.

**Figure 4.**
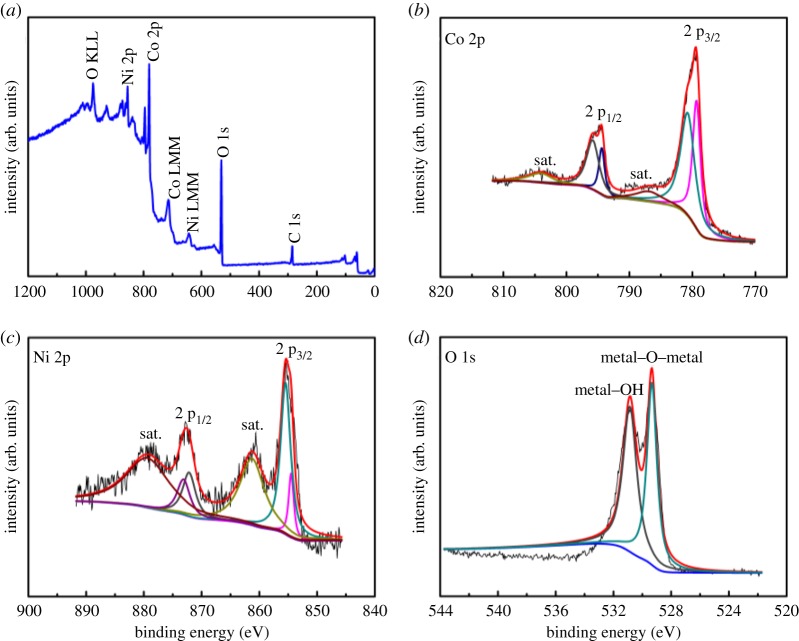
XPS spectra of Co_0.75_Ni_0.25_(OH)_2_: D: (*a*) wide scan spectrum, and high-resolution XPS spectra of (*b*) Co 2p, (*c*) Ni 2p and (*d*) O 1s regions.

To ascertain the chemical bonding states of every element in cobalt–nickel LDH, Co_0.75_Ni_0.25_(OH)_2_: D as a representative example was characterized by XPS analysis (figure 4). Figure 4*a* depicts the full spectrum of Co_0.75_Ni_0.25_(OH)_2_: D. The peaks can be identified as the contribution from Ni, Co, O and C, which manifest the existence of these elements in tested samples. The Co 2p spectrum (figure 4*b*) can be mainly split into two peaks centred at 794.9 eV for Co 2p_1/2_ and 779.3 eV for Co 2p_3/2_. Also, the spin–orbit splitting value of 15.6 eV and weak Co 2p_3/2_ satellite line intensity confirm the coexistence of Co^2+^ and Co^3+^ [37,38]. As exhibited in figure 4*c*, the peaks located at 872.5 eV for Ni 2p_1/2_ and 855.2 eV for Ni 2p_3/2_ accompanied with two shake-up satellites are the trait of Ni^2+^ [39,40]. The bands at 529.2 and 530.9 eV (figure 4*d*) result from O in metal–O–metal and metal–OH groups, respectively [41].

**Figure 5.**
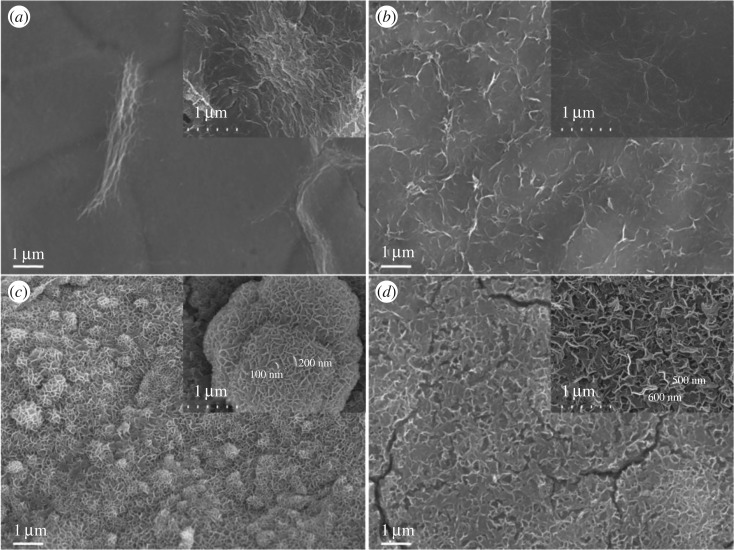
SEM images of Co_0.75_Ni_0.25_(OH)_2_ LDHs: (*a*) Co_0.75_Ni_0.25_(OH)_2_, (*b*) Co_0.75_Ni_0.25_(OH)_2_: H, (*c*) Co_0.75_Ni_0.25_(OH)_2_: D and (*d*) Co_0.75_Ni_0.25_(OH)_2_: C. Images of higher magnification are shown in respective insets.

**Figure 6.**
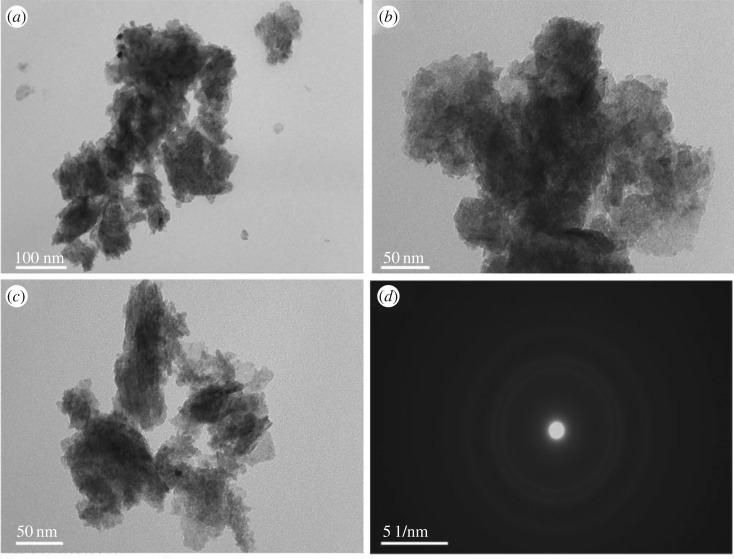
TEM images of Co_0.75_Ni_0.25_(OH)_2_ : D with different magnifications and locations (*a*–*c*), and the corresponding SAED pattern (*d*).

SEM and TEM provide further insight into the morphology and microstructure. As revealed by detailed morphologies in the images of figure 5, the surface morphology divergence indicates the function of surfactant during the synthesis process of electrodeposition. The Co_0.75_Ni_0.25_(OH)_2_ film obtained by electrodeposition without adding any surfactant exhibits a smooth surface with few wrinkled paper-like sheets covered on the nickel foam substrate (cf. Figure 5*a*). The wrinkles become more pronounced and uniformly continuous on the surface of Co_0.75_Ni_0.25_(OH)_2_: H electrode (figure 5*b*). Moreover, in figure 5*c* for the Co_0.75_Ni_0.25_(OH)_2_: D sample, a single nano-sheet is grown, and the nano-sheets form a cauliflower-like 3D structure by self-assembling simultaneously. The significant 3D pores cause a loosely packed structure, which further boosts electrolyte ions to access the active materials during the fast Faradaic reaction process [19]. The presence of crack-like flaws in the Co_0.75_Ni_0.25_(OH)_2_: C sample can be found in figure 5*d*; the structure of a serried net-like (the inset of figure 5*d*) interconnected surface with inter-flake pores offers passageway for intercalation and de-intercalation of ions and electrolyte diffusion in the bulk. The TEM characterization was done by separating the Co_0.75_Ni_0.25_(OH)_2_: D active material from Ni foam; there are some faint nano-sheets on the grid in figure 6*a*–*c* manifesting its ultrathin nature [18]. The corresponding selected area electron diffraction (SAED) pattern (figure 6*d*) possesses broad and diffused halo, suggesting its polycrystalline feature [42], which is well consistent with the result of XRD test.

### 3.3. Electrochemical performance of cobalt–nickel layered double hydroxides

**Figure 7.**
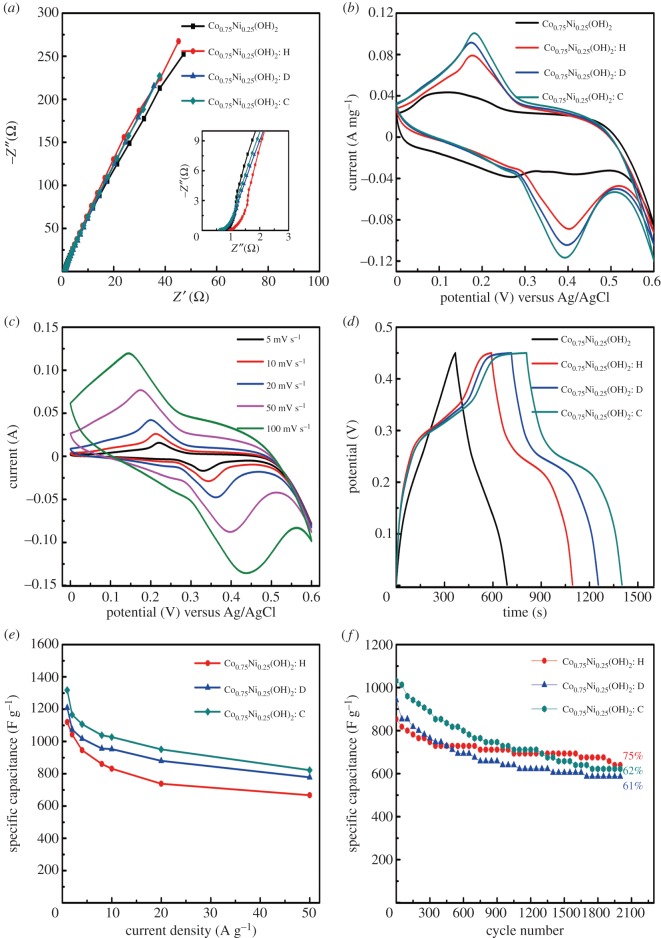
Electrochemical tests of electrodes synthesized without or with using surfactants: (*a*) Nyquist plots, (*b*) CV curves at a fixed scan rate of 50 mV s^−1^, (*c*) CV curves of the Co_0.75_Ni_0.25_(OH)_2_: D electrode at different scan rates, (*d*) galvanostatic charge–discharge curves at a fixed current density of 1 A g^−1^, (*e*) specific capacitance plots at different current densities and (*f*) cycling stabilities at a current density of 8 A g^−1^.

The electrochemical performances of the cobalt–nickel LDHs were evaluated by a typical three-electrode experimental cell equipped with a working electrode, a platinum foil counter electrode and an Ag/AgCl reference electrode. Electrochemical impedance spectroscopy (EIS) was employed in the frequency range from 100 kHz to 0.01 Hz to understand the electrochemical reaction kinetics of the as-synthesized samples. From figure 7*a*, it is apparent that the curves almost overlap; no significant differences of the Nyquist plots of the cobalt–nickel LDHs can be found. From the enlarged high-frequency region shown in the inset of figure 7*a*, by virtue of the binder-free and layered structure, no semicircles are visualized in this region, which is correlated to a low charge transfer resistance [43]. Figure 7*b* exhibits the cyclic voltammetry (CV) curves of these electrodes in 2 M KOH electrolyte at a fixed scan rate of 50 mV s^−1^ in the potential range of 0–0.6 V. The area of the CV curve of Co_0.75_Ni_0.25_(OH)_2_: C is larger than that of the others, manifesting better Sc. The typical CV curves of Co_0.75_Ni_0.25_(OH)_2_: D at different scan rates are displayed in figure 7*c*, where all the curves maintain the same shape with the change in scan rates. It is associated with the improved mass transportation [44]. It is easy to find out that the approximately same current responses of reduction and oxidation peaks are arising from the superb electrochemical reversibility. The redox peaks can be attributed to the following three faradic reactions [45]:

3.3 $$\text{Ni}{\left(\text{OH}\right)}_{2}+\text{O}{\text{H}}^{-}↔\text{NiOOH}+{\text{H}}_{2}\text{O}+{\text{e}}^{-},$$

3.4 $$\text{Co}{\left(\text{OH}\right)}_{2}+\text{O}{\text{H}}^{-}↔\text{CoOOH}+{\text{H}}_{2}\text{O}+{\text{e}}^{-}$$

3.5 $$\;\text{and}\;\text{CoOOH}+{\mathrm{OH}}^{-}↔\text{Co}{\text{O}}_{2}+{\text{H}}_{2}\text{O}+{\text{e}}^{-}.$$

To further explore the electrochemical performance of the cobalt–nickel LDH electrodes, the galvanostatic charge–discharge technique was performed at the current density of 1 A g^−1^ in the potential between 0 and 0.45 V. In figure 7*d*, all the charge–discharge curves have well-defined plateaus, which coincide with the result of CV test. The Sc of each specimen was determined from the galvanostatic charge–discharge curves by the following equation [46]:

3.6 $$C=\frac{I\Delta t}{m\Delta V},$$

where *C*, *I*, Δ*t*, *m*, Δ*V* refer to specific capacitance (F g^−1^), applied current load (A), discharging time(s) and mass of active material (g), potential window (V), respectively. The obtainable high values of Sc for Co_0.75_Ni_0.25_(OH)_2_: C (1318.2 F g^−1^), Co_0.75_Ni_0.25_(OH)_2_: D (1209.1 F g^−1^) and Co_0.75_Ni_0.25_(OH)_2_: H (1120 F g^−1^) electrodes at the current density of 1 A g^−1^ are in contrast to the low value for the pure Co_0.75_Ni_0.25_(OH)_2_ electrode (713.1 F g^−1^). It is thus evident that 3D structure fabricated through the use of surfactant promotes the electrochemical performance. Figure 7*e* displays the Scs of the cobalt–nickel LDHs synthesized with surfactants when the charge–discharge rate increases from 1 to 50 A g^−1^. The electrode of Co_0.75_Ni_0.25_(OH)_2_: D retains about 64% of the Sc at 50 A g^−1^, while 62% and 57% capacitance retentions for Co_0.75_Ni_0.25_(OH)_2_: H and Co_0.75_Ni_0.25_(OH)_2_: C electrodes are observed, respectively. These consequences imply that the electrodes of cobalt–nickel LDHs possess high rate capabilities. Figure 7*f* depicts the cycling stability tested at a current density of 8 A g^−1^. Seventy-five per cent of initial Sc is kept after 2000 cycles for Co_0.75_Ni_0.25_(OH)_2_: H, while 62% for Co_0.75_Ni_0.25_(OH)_2_: C and 61% for Co_0.75_Ni_0.25_(OH)_2_: D are retained.

### 3.4. Electrochemical performance of cobalt–nickel layered double hydroxides//activated carbon asymmetric supercapacitors

The practicability of these materials was investigated by assembling ASCs using the cobalt–nickel LDHs as positive electrodes and AC as negative electrodes in 6M KOH aqueous electrolyte. It is found that the optimal mass ratio to fabricate the ASC device is *m*_ac_/*m*_cobalt nickel LDH_ = 2.4 according to the following equation [47]:

3.7 $$\frac{m_{+}}{m_{-}}=\frac{c_{-}\Delta V_{-}}{c_{+}\Delta V_{+}},$$

where *m_+_* and *m_−_* are the mass of electrode materials, *c*_+_ and *c*_−_ are the specific capacitances of the positive and negative electrodes, Δ*V_+_* and Δ*V_−_* represent the potential window for the electrodes, respectively.

**Figure 8.**
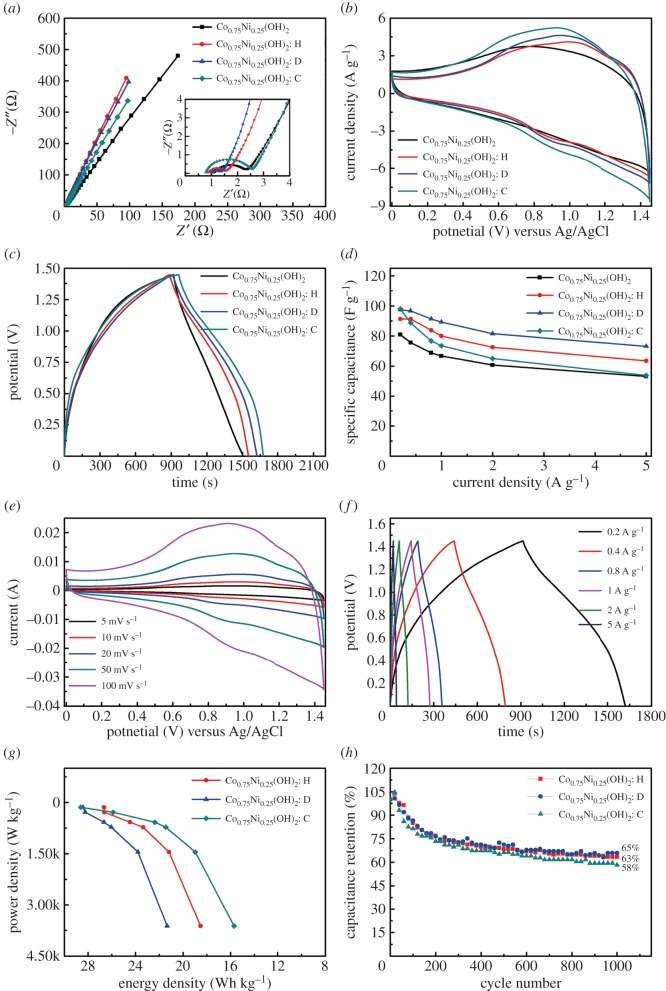
Electrochemical evaluation of Co_0.75_Ni_0.25_(OH)_2_ LDHs//AC ASCs: (*a*) EIS spectra, (*b*) CV curves at a scan rate of 50 mV s^−1^, (*c*) galvanostatic charge–discharge curves at a current density of 0.2 A g^−1^, (*d*) specific capacitance at different current densities, (*e*) CV curves of the Co_0.75_Ni_0.25_(OH)_2_: D//AC at various scan rates, (*f*) galvanostatic charge–discharge curves of the Co_0.75_Ni_0.25_(OH)_2_: D//AC at different current densities, (*g*) Ragone plot for energy density and power density and (*h*) cycle stabilities of ASC devices at a current density of 0.8 A g^−1^.

As can be seen from figure 8*a*, all EIS plots of these ASCs contain a semicircle in the high-frequency region and then a straight sloping line; the smallest diameter of the semicircle of Co_0.75_Ni_0.25_(OH)_2_: D//AC is associated with a smallest charge transfer resistance (*R*_CT_) [48]. The low *R*_CT_ is pretty vital to high electrical conductivity and outstanding rate capability [48]. Benefiting from the pseudo-capacitance and electrical double-layer capacitance, the CV curves shown in figure 8*b* were obtained in the window of 0–1.45 V at a scan rate of 50 mV s^−1^. The CV curves of the Co_0.75_Ni_0.25_(OH)_2_: D//AC keep the same shape at various scan rates, which means the fast charge–discharge property of the device (figure 8*e*). From the galvanostatic charge–discharge curves at the current density of 0.2 A g^−1^ (figure 8*c*), the discharge time of Co_0.75_Ni_0.25_(OH)_2_//AC is shortest among these ASCs. Energy density (*E*) and power density (*P*) were estimated using the following equations [48]:

3.8 $$E=\frac{1}{2}C\Delta V^{2}$$

and

3.9 $$P=\frac{E}{\Delta t}\;\times \;3600.$$

The energy densities of the devices can be further calculated to be 28.6 Wh kg^−1^ (Co_0.75_Ni_0.25_(OH)_2_: C//AC), 28.4 Wh kg^−1^ (Co_0.75_Ni_0.25_(OH)_2_: D//AC) and 26.7 Wh kg^−1^ (Co_0.75_Ni_0.25_(OH)_2_: H//AC). This result further implies that the use of surfactant during the electrodeposition process is advantageous to the electrochemical performance. As the current density increases from 0.2 to 5 A g^−1^ shown in figure 8*f*, the calculated Sc value of the Co_0.75_Ni_0.25_(OH)_2_: D//AC ASC device decreases from 97.3 to 73.1 F g^−1^. As shown in figure 8*d*, the trend in rate capabilities of Co_0.75_Ni_0.25_(OH)_2_: D//AC (75%), Co_0.75_Ni_0.25_(OH)_2_: H//AC (69.3%), Co_0.75_Ni_0.25_(OH)_2_//AC (65%) and Co_0.75_Ni_0.25_(OH)_2_: C//AC (54.8%) is similar to the trend in the diameters of the semicircles in the inset of figure 8*a*. This observation supports the fact that Co_0.75_Ni_0.25_(OH)_2_: D//AC has better interfacial faradic reaction kinetics; the Co_0.75_Ni_0.25_(OH)_2_: D//AC device also keeps a better cycle life (65%) after 1000 cycles in figure 8*h*. The two important results are owing to the preferable electronic conductivity and unique surface structure; the cauliflower-like structure of Co_0.75_Ni_0.25_(OH)_2_: D has many interspaces between the particles, which allow the volume expansion to take place easily, and promote the high distribution of active sites as well as high speed pathway for continuous electronic and ionic transportation [37]. Ragone plots of the ASCs are shown in figure 8*g*. A maximum power density (3625 W kg^−1^) at an energy density of 21.3 Wh kg^−1^ was achieved by the Co_0.75_Ni_0.25_(OH)_2_: D//AC ASC device, which is in line with the conclusion that a high rate ability of the electrode material favours supercapacitor providing a high power density [49]. Although the Co_0.75_Ni_0.25_(OH)_2_: D//AC electrode does not present the highest Sc, it still seems more suitable for practical application on grounds of its preferable rate capability and cycle life.

## 4. Conclusion

The morphologies of cobalt–nickel LDHs were successfully modified by using surfactants during the process of cathodic electrodeposition. In comparison to the pristine Co_0.75_Ni_0.25_(OH)_2_ active material having a Sc of 713.1 F g^−1^, the modified Co_0.75_Ni_0.25_(OH)_2_ LDHs with distinct surface morphologies showed superior Scs: wrinkle-like, cauliflower-like and net-like Co_0.75_Ni_0.25_(OH)_2_ electrodes exhibited Sc of 1120 F g^−1^, 1209.1 F g^−1^ and 1318.2 F g^−1^, respectively. Among ASC devices fabricated using Co_0.75_Ni_0.25_(OH)_2_ LDHs as positive electrodes, the Co_0.75_Ni_0.25_(OH)_2_: D//AC ASC device showed a preferable energy density at the same power density. These results demonstrated that morphology modification by surfactant enhanced the electrochemical performance in cobalt–nickel LDH electrode materials.

## Data accessibility

Our data collected and analysed for this study have been deposited with Dryad and can be accessed at Dryad Digital Repository: http://dx.doi.org/10.5061/dryad.61284n2 [50].
